# Connecting perceived economic threat and prosocial tendencies: The explanatory role of empathic concern

**DOI:** 10.1371/journal.pone.0232608

**Published:** 2020-05-04

**Authors:** María Alonso-Ferres, Ginés Navarro-Carrillo, Marta Garrido-Macías, Eva Moreno-Bella, Inmaculada Valor-Segura

**Affiliations:** 1 Department of Social Psychology, University of Granada, Granada, Spain; 2 Department of Psychology, University of Jaén, Jaén, Spain; Middlesex University, UNITED KINGDOM

## Abstract

Recent research suggests that perceived economic threat constitutes a valid predictor of people’s attitudes and behaviors. While accumulated empirical evidence has mostly underlined the deleterious psychological effects (e.g., reduced psychological well-being) of perceived economic threat in times of economic strain, we postulate that individuals experiencing higher economic threat linked to the Spanish economic crisis are more prone to engage in other-beneficial prosocial behavior. Across two independently collected community samples, we tested this theoretical formulation and examined the potential mediating roles of empathic concern (Studies 1 & 2) and identification (Study 2). Study 1 (*N* = 306) revealed that participants who descended in the social scale due to the negative national economic context were engaged in a larger number of helping behaviors over the last three months compared to participants who did not descend the social ladder—independently of several sociodemographic and ideological factors. Moreover, our data indicated these effects were driven by increased empathic concern. Study 2 (*N* = 588), in which two hypothetical helping-behavior scenarios were randomly administered (crisis-related vs. control), showed that participants under high perceived financial threat exhibited an undifferentiated pattern of prosociality. However, moderated-mediation analyses indicated that empathic concern explained the perceived financial threat-helping behavior link in the hypothetical crisis-related scenario but not in the hypothetical control scenario. Together, these findings extend prior literature on the psychosocial effects of perceived economic threat and the determinants of other-oriented behavior. Implications of these findings and suggestions for further research are discussed.

## Introduction

The recent global economic crisis entails the more dramatic period of economic decline that several countries around the world have gone through over the past decades. This economic crisis, commonly referred to as the Great Recession, had its origin in the United States between the end of the 2007 and the beginning of 2008 [1,2]. This context of economic instability substantially impacted not only the United States—unemployment, inequality, and poverty rates increased considerably within a relatively short space of time [3,4]—but also various industrialized European regions that in the pre-crisis phase enjoyed robust economic growth. The case of Spain is, in this sense, paradigmatic, given that its economy experienced a lengthy expansive cycle in the years preceding the onset of the economic crisis [5]. Nonetheless, with the crisis, the Spanish general economic background indicators significantly worsened [6]. Nearly a decade after the outbreak of the crisis, the Spanish socioeconomic reality is still suffering its effects; for instance, the unemployment rate is about double what it used to be [7], the public debt is almost 100% of the Gross Domestic Product (GDP) [8], and economic inequality and poverty are becoming increasingly chronic [9].

Broadly speaking, the data above highlight the need for further examination of potential wide-ranging implications associated with tough economic climates. Empirical research has mainly focused on investigating the socioeconomic and political impacts of the global economic downturn [10–13], while analysis of its psychological repercussions has captured relatively little scholarly attention. Overall, within this framework, most studies have given a special focus to the effects of the economic crisis on psychological well-being and health-related indicators. For example, documentation has linked this period of economic downturn to reduced life satisfaction [14], poorer mental health and greater work-related stress [15], and lower levels of self-efficacy [16], personal control [17], and general trust [18]. Notwithstanding these detrimental psychological outcomes, it has been suggested that those most affected by economic crises tend to establish networks of mutual solidarity and social support as a plausible strategy for coping with adverse personal and socioeconomic circumstances [19]. Following this suggestion, we intended in our research to contribute to better understanding of the various psychological implications of economic hard times by exploring whether the perceived personal impact of the Spanish economic crisis is connected to prosocial behavior. Specifically, our research aims to test the hypothesis that a higher perceived economic threat linked to the economic downturn is associated with a greater inclination to engage in other-beneficial prosocial behavior. Additionally, we theorize that empathic concern acts as a psychological mechanism through which the state of being affected by a negative national economic situation affects prosociality.

### Perceived economic threat and prosocial behavior

Current psychological approaches suggest that times of economic turmoil and rising social inequality clearly exemplify sources of economic threat that could instigate destructive or potentially negative psychological responses [20]. For instance, recent research indicates that increased income inequality, one of the most distinctive features of the Great Recession [21], may exacerbate social distance between citizens [22], thereby fostering individualistic tendencies [23] and perceptions of interpersonal competitiveness [24]. Nevertheless, research that directly analyzes the links between the experience of personal economic threat related to the economic crisis and theoretically relevant psychological outcomes is limited [25]. In spite of this, some valuable empirical data are available. In this vein, Becker, Wagner, and Christ [26] indicated using a representative German survey that personal threat triggered by the financial crisis was positively related to ethnic prejudice. Along the same lines, Fritsche et al. [17], using a Spanish general community sample and a randomized, representative face-to-face German survey, found that perceived personal deterioration and fear of economic decline on the social scale predicted higher in-group bias and hostile interethnic attitudes (i.e., chauvinism and xenophobia), respectively. Even though it seems that there is increasing consensus on the notion that personal economic threat related to the economic crisis may engender predominantly disruptive psychological responses, we consider the reverse notion also plausible; that is, suffering the effects of the economic crisis could be associated with positive and constructive interpersonal responses. In particular, we surmise that feeling personally threatened in times of economic hardship might be connected with a higher inclination to engage in prosocial behavior.

Why might economic threat cause people to be more prosocial? One possibility for individuals moving to prosocial behavior is the intent to alleviate unpleasant, threatened feelings stemming from an unfavorable economic environment. Specifically, people who show higher levels of perceived threat due to situations of economic disturbance (e.g., economic crisis) may experience reduced control over their environments [17] and increased vulnerability or sensitivity to these sources of threat. One way to withstand personal threats is to engage in other-oriented tendencies (i.e., prosocial behavior) that allow individuals to build and sustain cooperative networks to provide and receive joint protection during tough times [27,28]. Indeed, it is theorized that social contacts or attachment-related behaviors can protect against the adverse effects of falling under threats [29].

Although there is no direct evidence linking perceived economic threat related to the crisis with increased prosocial behavior, prior research provides indirect support for this notion. For example, individuals from lower-socioeconomic backgrounds were found more prone to engage in prosocial behavior relative to their counterparts from upper-socioeconomic levels [27]. Studies on cooperation across cultures also lend credence to this claim. An American national research survey [30] found that higher-income people spend a small proportion of their money assisting others in need. Conversely, lower-income individuals give proportionally more of their incomes to helping others in need [31,32]. Likewise, in 2014, a cross-cultural study of 27 nations showed that people with fewer socioeconomic resources approved fewer unethical actions compared to their counterparts of higher socioeconomic positions [33]. Investigations conducted with individuals who have suffered from threatening experiences are also in keeping with this idea. Researchers have concretely shown that past adversity is associated with a higher tendency to assist others (e.g., helping a stranger) [34,35].

Within the body of research examining the effects of the economic downturn on prosocial behavior, the scarce empirical evidence that exists has revealed that communities hit by the American foreclosure crisis volunteered at increasing rates [36]. Also aligning with our main theoretical formulation, albeit focusing on the Spanish context of unemployment, Bukowsky, de Lemus, Rodríguez-Bailón, Willis, and Albuquerque [37] consistently found that salient lack of personal control leads to more positive intergroup evaluations. Although preceding findings are certainly suggestive, the present research directly extends these prior works by analyzing differences in various measures of prosociality based on people’s levels of perceived economic threat. To the best of our knowledge, no studies have directly tested this association in the context of the Spanish economy, which has been among the ones affected the worst by the Great Recession.

### Perceived economic threat, concern for others, and prosocial behavior

Empathic concern—an affective state that stems from the ability to judge and adopt the emotions of other individuals [38,39]—is an important predictor of relationship outcomes and social adjustment [40]. Interestingly, different studies have argued that empathic concern is shaped by different characteristics of the social context, ranging from cultural dimensions to social roles or socioeconomic position [41–43]. However, no empirical investigations have explored the consequences on empathic concern of experiencing effects of economic crisis. We posit that exposure to stressful situations could also increase people’s emotional sensitivities toward others’ needs as a way to mitigate personal threat perception. We draw our assumptions from Taylor’s theory [28]. This theoretical model proposes that one way of coping with stressful environments is to engage in affiliative behaviors, thereby building cooperative networks that may facilitate the struggle against external threats. This affiliative response strategy is theorized to foster greater attention to the needs of others, and to result in more prosocial responses to suffering. Indeed, some studies have suggested that negative feelings deriving from social and interpersonal problems may make individuals more sensitive to the plights of others because of their increased understandings of negative events, which ultimately foster empathic concern tendencies [44–46]. For example, Kraus et al. [41] found that individuals belonging to a lower socioeconomic status were more attentive to others’ emotional states, showing greater empathic accuracy than those belonging to an upper socioeconomic status. Likewise, lower-class individuals have been reported to display different types of empathy-related responses to others’ suffering (e.g., heart rate deceleration or fronto-central P2 responses) to a greater extent than upper-class individuals [47,48]. Therefore, one might expect individuals’ levels of economic threat to affect prosocial emotions, such as empathic concern.

Empathic concern could in turn be related to a greater inclination to express prosocial behaviors among those individuals most affected by the economic crisis. Indeed, the positive effect of empathic concern—measured as both a state and trait—on prosocial behavior has been widely accepted among several psychological experimental and non-experimental studies [38, 49–53]. For instance, empathic concern has been linked to higher levels of self-reported charitable giving [54]. Recently, Klimecki, Mayer, Jusyte, Scheeff, and Schönenberg [55] showed higher levels of altruistic behavior and more than 40% of the increase in sharing behavior among participants who were induced to feel concern for one another’s welfare. Moreover, Weng, Fox, Hessenthaler, Stodola, and Davidson [56] posited that feelings of empathic concern toward those in need are associated with action to help those individuals. Additionally, Lim and DeSteno [34] found a robust link between empathic concern toward individuals’ suffering and the behavioral responses (i.e., charitable giving, helping a stranger) taken to assist them, especially noticeable in participants who had experienced past adversity.

Overall, the available findings provide indirect support for the contention that perceived economic threat linked to the crisis could be associated with greater empathic concern, which in turn would be positively connected to prosocial behavior.

## Study 1

The purpose of the first study was to empirically test the hypothesis: the higher the perceived personal impact of the economic crisis, the greater is the willingness to engage in prosocial behavior. Considering that prior research has shown gender, objective and subjective socioeconomic status, and political orientation to be related to other-oriented emotions and behaviors [27, 57–59], we included them as control variables in the analytical models. We also explored whether feeling impacted by a negative economic context affected prosocial behavior even after accounting for employment status. Lastly, the current study was also aimed at elucidating whether empathic concern may act as a mediating factor within the perceived impact-prosocial behavior relationship.

### Method

#### Participants

The sample was composed of 306 participants (*M*_age_ = 28.38, *SD* = 10.83, range from 17 to 65), of which 59.1% were women and 40.9% were men (see more sociodemographic characteristics in Table 1). A sensitive power analysis was conducted using a MANCOVA special effects and interactions test [60] to determine our ability to detect the contribution of a between-subject MANOVA. Given our sample (*N* = 306, α = 0.05), sensitivity analysis suggests the need for a minimum effect size of *f*^2^ ≥ .02 to produce power at the 0.80 level.

**Table 1:** Sociodemographic information of participants in studies 1 and 2.

Variable	Study 1 (*N* = 306)		Study 2 (*N* = 588)	
	*n*	%	*n*	%
**Civil status**				
Single	143	46.7	161	27.4
Married	50	16.3	186	31.6
Divorced	6	2.0	29	4.9
Involved in a relationship (dating or cohabiting)	95	31.0	212	36.1
Not reported	12	3.9	-	-
**Currrent employment situation**				
Student	-	-	93	15.8
Full-time job	105	34.3	178	30.3
Part-time job	46	15	130	22.1
Retired	2	.7	9	1.5
Un-employed	139	45.4	178	30.3
Not reported	14	4.6	-	-
**Family income level**				
< 650€	12	3.9	58	9.9
651€–1300€	51	16.7	212	36.1
1301€–1950€	65	21.2	161	27.4
1951€–2600	56	18.3	86	14.6
2601€–3250€	36	11.8	34	5.8
3251€–3900€	22	7.2	18	3.1
3901€–4550€	17	5.6	8	1.4
4551€–5200€	8	2.6	3	0.5
5201€–5800€	4	1.3	4	0.7
> 5800€	10	3.3	4	0.7
Not reported	25	8.2	-	-
**Educational attainment**				
Primary school	12	3.9	32	5.4
Secondary education	11	3.6	54	9.2
Vocational training	29	9.5	116	19.7
Bachelor	28	9.2	54	9.2
University not completed	91	29.7	84	14.3
University completed	56	18.3	143	24.3
Master	42	13.7	97	16.5
Doctorate	21	6.9	8	1.4
Not reported	16	5.2	-	-

#### Procedure

Three previously trained evaluators requested potential volunteers to participate in different public areas (e.g., local transport stations, particularly the rest area of the bus station) located in Granada, a southeast Spanish city strongly affected by the financial crisis. Specifically, by means of an incidental sampling procedure, the evaluators first approached potential participants in the rest area of local transport stations. Next, they identified themselves as researchers in psychology and asked participants to complete a written questionnaire about their beliefs on some social issues. Moreover, evaluators informed respondents about the estimated duration to fill in the booklet (approximately 15 min) and their voluntary participation. The anonymity and confidentiality of the participants’ answers were also emphasized. As selection criterion, participants were required to have Spanish as their native language, due to the questionnaire’s being presented to them in such language. Once participants accepted to participate in the study, they were instructed to complete the questionnaire as accurately as possible and told that they could drop the study if they decided to do so. After that, participants gave informed written consent in accordance with the Declaration of Helsinki and, subsequently, completed the study booklet (in the order described below) under the supervision of the researchers. Finally, volunteers were thanked and debriefed and did not receive any type of financial or material compensation for their participation. The study received approval from the institutional research ethics committee of the University of Granada (Number: 1002/CEIH/2019).

#### Measures

*Helping behavior*. To measure helping behavior, we used the “Helping Behavior in Everyday Life” scale [61]. This instrument comprises 13 items assessing formal planned helping (e.g., “Donated blood or any other medical item”), 18 items evaluating informal planned helping (e.g., “Helped someone move into a house”), and 15 items measuring spontaneous helping (e.g., “Delayed an elevator or held the elevator door open for someone who wanted to get in”). Participants indicated how many of the activities described in the scale they had engaged in over the last three months. The items used a dichotomous answer format (0 = no, 1 = yes), and we operationalized helping behavior as the sum of answers to the items.

*Empathic concern*. We used the empathic concern measure included in the Interpersonal Reactivity Index (IRI) [62,63]. Participants indicated their levels of agreement with its eight items (e.g., “I am often quite touched by things that I see happen”) on a 5-point Likert scale with scores ranging from 1 (*strongly disagree*) to 5 (*strongly agree*); α = .76.

*Perceived economic threat related to the economic crisis*. We evaluated whether or not participants had fallen in the social scale as a result of the Spanish economic crisis by administering a measure recently used by prior psychological research [17,18] and socioeconomic surveys [64]: “Faced with the current economic situation and thinking about your and your family’s situation, do you believe that economic crisis has made you descend in the social scale?”: (1) *Yes*, *I used to be in the upper class*, *and now I am in the upper middle class;* (2) *Yes*, *I used to be in the upper middle class*, *and now I am in the middle class;* (3) *Yes*, *I used to be in the middle class*, *and now I am in the lower middle class;* (4) *Yes*, *I used to be in the lower middle class*, *and now I am in the lower class;* (5) *Yes*, *I used to be in the lower class*, *and now I am in a very delicate situation*, *dreading a fall into poverty;* (6) *No*, *the crisis has not made me descend in the social scale;* (7) *No*, *the crisis has made me ascend in the social scale;* and (8) *I prefer not to answer*. We created two groups based on participants’ responses, one group including participants who had descended in the social hierarchy (responses 1–5; 38.8%) and the other consisting of participants who had not descended in the social hierarchy (responses 6–7; 60.5%). Perceived socioeconomic decline is considered a relevant indicator of personal economic threat [20].

*Subjective social class*. Participants’ subjective social classes were assessed by administering the MacArthur Scale of Subjective Social Status [65]. Participants were presented a ladder with 10 rungs representing different levels of income, education, and occupation status in society. They were asked to indicate the rungs they think they stand on within the ladder. High numbers indicated higher placements on the social ladder.

*Objective social class*. For consistency with prior studies [66,67], objective social class was operationalized as the summation of standardized scores on participants’ family income and educational attainment.

*Political orientation*. Participants indicated their political orientations on a scale ranging from 1 (*left-wing*) to 10 (*right-wing*).

#### Statistical analyses

Descriptive statistics and partial (controlling for gender) bivariate associations among tested variables were first computed. Then, to examine the effect of perceived economic threat linked to the crisis (1 = non-decline, 2 = socioeconomic decline) on helping behavior (i.e., formal planned behavior, informal planned behavior, and spontaneous helping), a between-subject MANCOVA analysis was conducted, controlling for the influence of sociodemographic and ideological characteristics (i.e., gender, objective and subjective socioeconomic status, employment status, and political orientation). Finally, we further examined the indirect effect of perceived economic threat on helping behavior parallel to the empathic concern. Three separate analyses of mediation for each type of helping behavior (i.e., formal planned behavior, informal planned behavior, and spontaneous helping behavior) were performed using Hayes’ [68] PROCESS macro (Model 4). Gender, objective and subjective socioeconomic status, employment status, and political ideology were included as covariates. Bias-corrected confidence intervals for indirect associations were estimated based on 5,000 bootstrap samples. Confidence intervals that do not contain zero indicate that effects are significant (*p* < .05).

### Results

#### Descriptive statistics and inter-correlations

Table 2 displays means, standard deviations, and partial product-moment coefficient correlations (controlling for gender) for tested variables. Participants who felt more affected by the Spanish economic downturn reported greater scores on empathic concern. They also exhibited greater formal planned helping and spontaneous helping behavior. Furthermore, higher empathic concern was associated with increased formal, informal, and spontaneous helping behaviors. All measures of helping behavior were positively correlated with each other. Additionally, objective and subjective socioeconomic status, which were positively correlated, were negatively associated with perceived economic threat. Lastly, political orientation was positively correlated with subjective socioeconomic status and negatively correlated with perceived economic threat, empathic concern, and formal planned helping. The correlations among the study variables were significant but lower than .70, so that there were no multicollinearity concerns.

**Table 2:** Descriptive statistics and correlations among study 1 variables.

	1	2	3	4	5	6	7	8
1. Perceived impact of EC	-							
2. Empathic concern	.17**	-						
	[0.05, 0.28]							
3. Formal PH	.16**	.27***	-					
	[0.04, 0.29]	[0.17, 0.37]						
4. Informal PH	.11	.16**	.43***	-				
	[-0.20, 0.23]	[0.05, 0.18]	[0.33, 0.52]					
5. Spontaneous helping	.15*	.23***	.48***	.64***	-			
	[0.03, 0.27]	[0.10, 0.34]	[0.38, 0.57]	[0.56, 0.71]				
6. Objective SES	-.26***	.02	.11	.06	.05	-		
	[-0.37, -0.14]	[-0.09, 0.14]	[-0.01, 0.23]	[-0.06, 0.18]	[-0.06, 0.18]			
7. Subjective SES	-.23***	.00	.04	-.09	-.04	.40***	-	
	[-0.34, -0.11]	[-0.12, 0.15]	[-0.08, 0.16]	[-0.22, 0.03]	[-0.17, 0.10]	[0.30, 0.50]		
8. Political Orientation	-.16**	-.20**	-.13*	-.02	-.10	.11	.28***	-
	[-0.27, -0.05]	[-0.32, -0.07]	[-0.25, -0.01]	[-0.13, 0.10]	[-0.23, 0.03]	[-0.00, 0.22]	[0.16, 0.39]	
*M*	1.38	3.83	3.02	8.62	6.33	-	5.82	4.18
*SD*	0.49	0.63	2.02	3.75	2.82	-	1.50	2.00

*N* = 306; EC: Economic crisis; PH: Planned helping. SES: Socioeconomic status; Gender (1 = males, 2 = females); Employment status (1 = no unemployed, 2 = unemployed). Higher scores on continuous variables indicate greater standing on the variable (e.g., greater empathic concern).

*p < .05,

**p < .01,

***p < .001

#### Effects of perceived economic threat on helping behavior indicators

**Fig 1:**
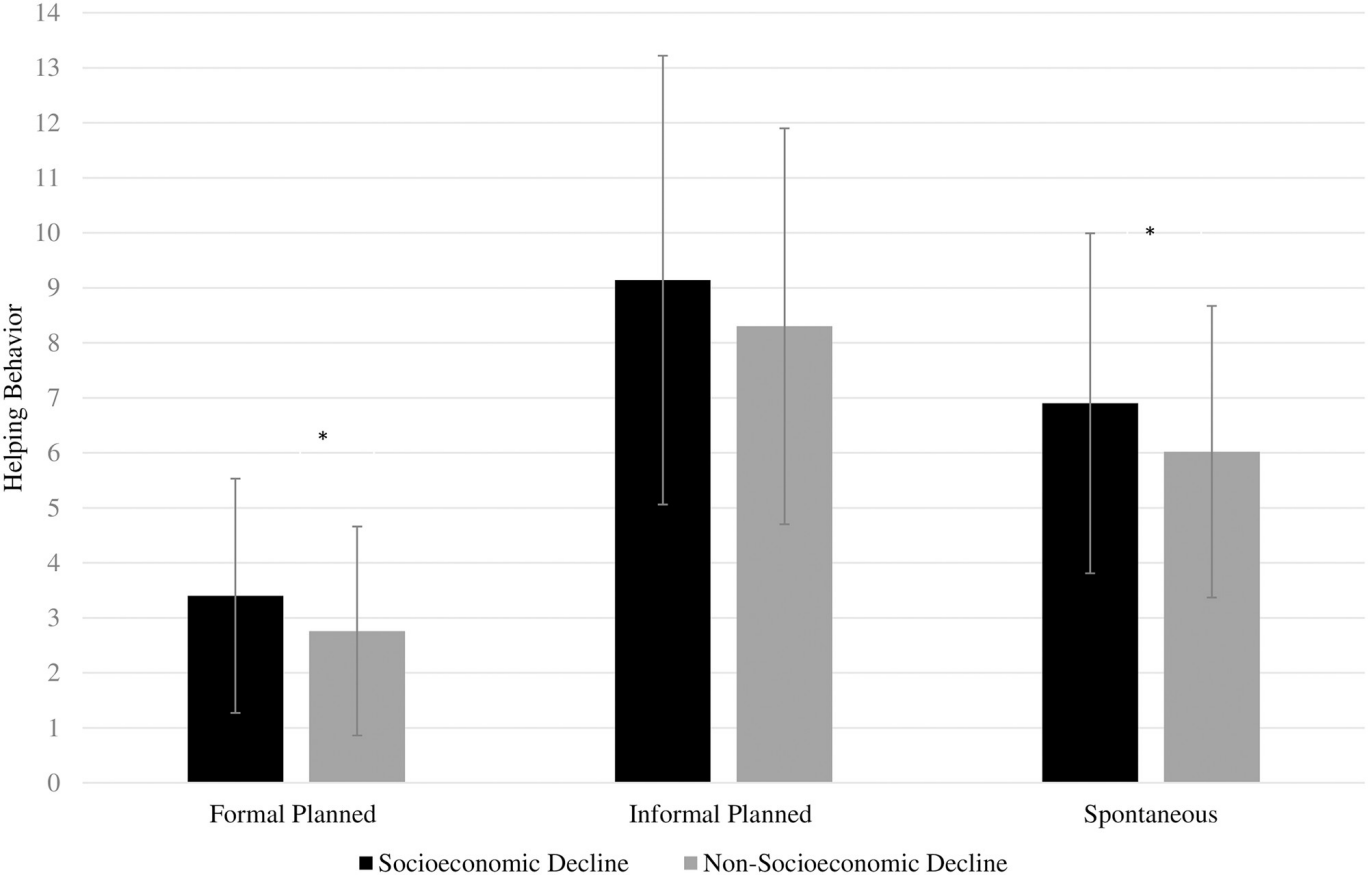
Helping behavior in individuals who descended in the social ladder as a result of the economic crisis (socioeconomic decline group) compared to individuals who did not descend in the social ladder (non-socioeconomic decline group).

As expected, a significant effect of perceived economic threat emerged, *Wilks’ λ* = .96, *F*(3, 264) = 3.79, *p* = .011, *η*^*2*^ = .04; in particular, individuals differed on their formal planned helping, *F*(1, 266) = 10.02, *p* = .002, *η*^*2*^ = .04, and spontaneous helping, *F*(1, 266) = 6.36, *p* = .012, *η*^*2*^ = .02. As Fig 1 displays, individuals who descended the social ladder due to the economic crisis (the socioeconomic decline group) showed greater scores on formal planned helping and spontaneous helping than individuals who did not descend the social ladder. No significant differences were found for informal planned helping, *F*(1, 266) = 3.45, *p* = .065, *η*^*2*^ = .01.

Differences in helping behavior were not observed in terms of gender, *Wilks’ λ* = .98, *F*(3, 264) = 1.09, *p* = .355, *η*^*2*^ = .01, objective socioeconomic status, *Wilks’ λ* = .98, *F*(3, 264) = 2.13, *p* = .097, *η*^*2*^ = .02, subjective socioeconomic status, *Wilks’ λ* = .98, *F*(3, 264) = 2.32, *p* = .076, *η*^*2*^ = .03, employment status, *Wilks’ λ* = .98, *F*(3, 264) = 1.84, *p* = .141, *η*^*2*^ = .02, or political orientation, *Wilks’ λ* = .98, *F*(3, 264) = 2.26, *p* = .082, *η*^*2*^ = .03.

#### Indirect effect of perceived economic threat on helping behavior indicators via empathic concern

**Fig 2:**
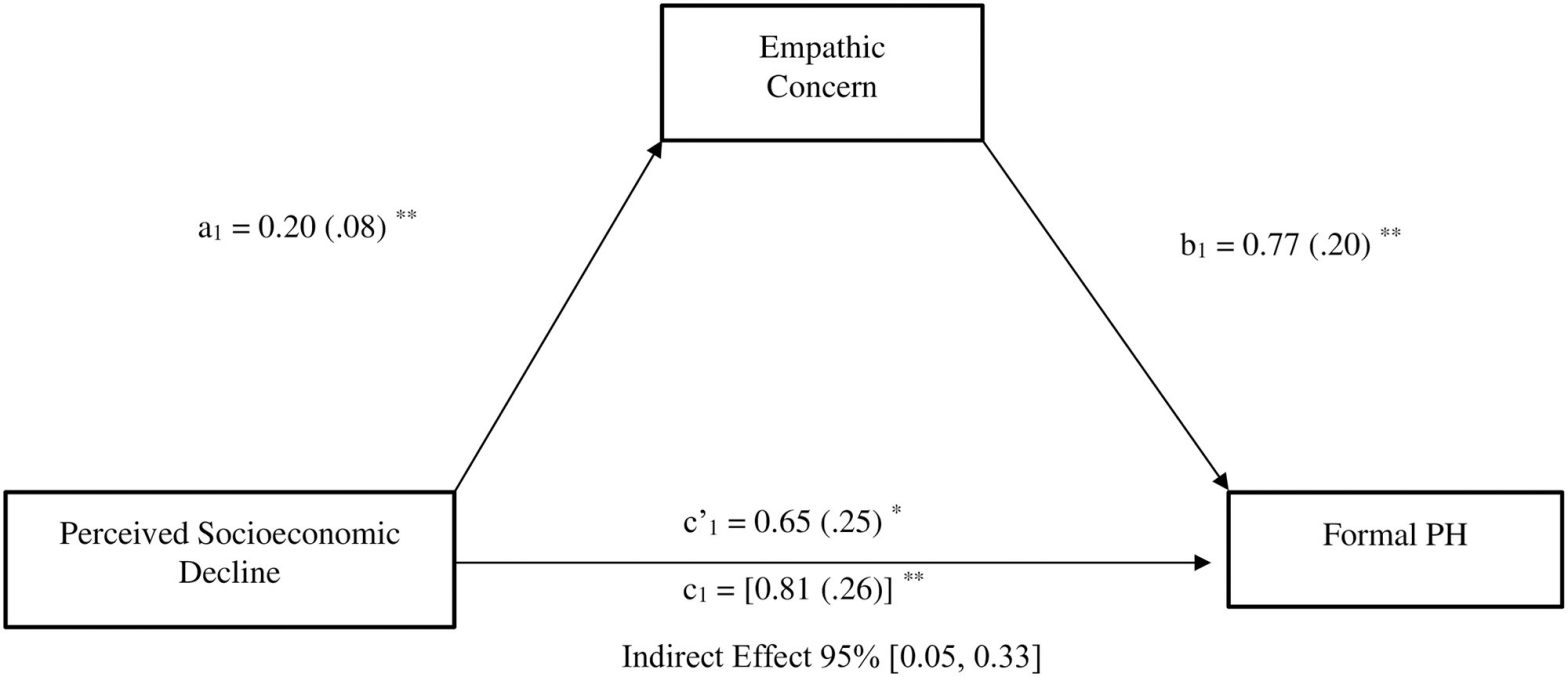
Mediation model depicting the indirect effect of perceived socioeconomic decline (1 = non-decline, 2 = decline) on formal Planned Helping (PH) behavior through empathic concern. Study 1; N = 306. All reported values are unstandardized estimates (b values), with their SE reported between parentheses. Bootstrap simple size: 5,000. The total effect of perceived socioeconomic decline on formal PH appears within brackets. *p < .05, **p < .01, ***p < .001.

Aligning with our expectations, results showed that perceived economic threat was indirectly linked to formal planned helping (*b* = .16, *SE* = .07, 95%CI [0.05, 0.33]), via the effect of perceived economic threat on empathic concern (see Fig 2).

**Fig 3:**
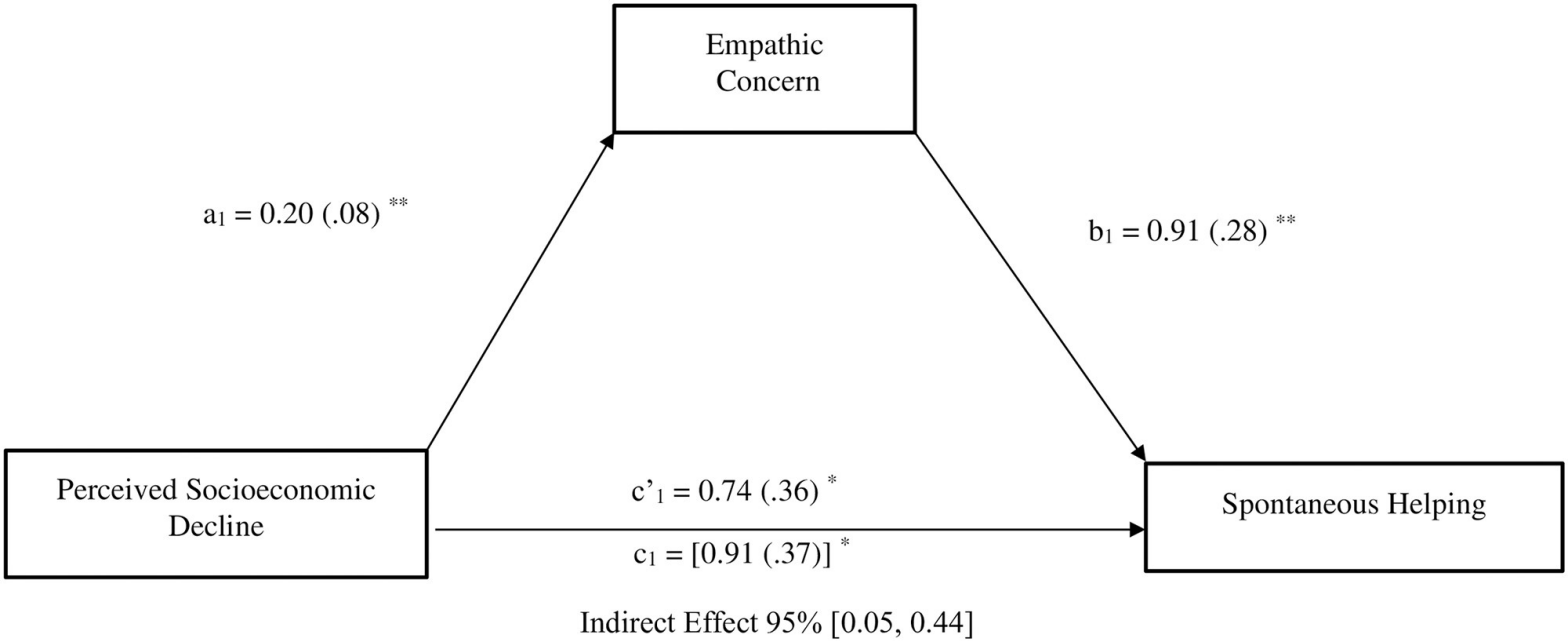
Mediation model depicting the indirect effect of perceived socioeconomic decline (1 = non-decline, 2 = decline) on spontaneous helping behavior through empathic concern. Study 1; *N* = 306. All reported values are unstandardized estimates (*b* values), with their SE reported between parentheses. Bootstrap simple size: 5,000. The total effect of perceived socioeconomic decline on spontaneous helping appears within brackets. *p < .05, **p < .01, ***p < .001.

Moreover, perceived economic threat was also indirectly linked to spontaneous helping (*b* = .19, *SE* = .09, 95%CI [0.05, 0.44]) via its effect on empathic concern (see Fig 3). After controlling for the effects of empathic concern, the direct effect of perceived economic threat on formal planned (*b* = .65, *SE* = .25, *p* = .011, 95%CI [0.15, 1.16]) and spontaneous helping remained significant (*b* = .74, *SE* = .36, *p* = .044, 95%CI [0.02, 1.44]), indicating the existence of partial mediations.

**Fig 4:**
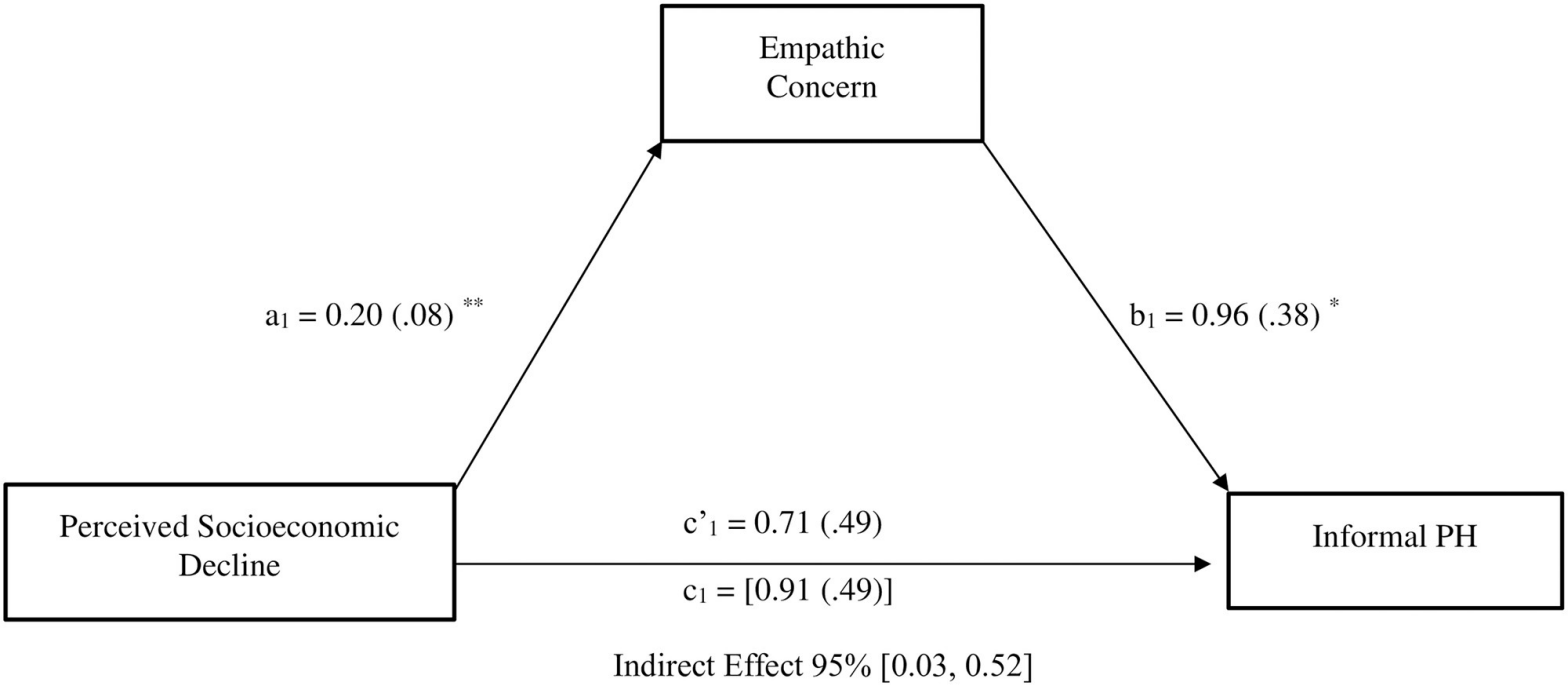
Mediation model depicting the indirect effect of perceived socioeconomic decline (1 = non-decline, 2 = decline) on informal Planned Helping (PH) behavior through empathic concern. Study 1; *N* = 306. All reported values are unstandardized estimates (*b* values), with their SE reported between parentheses. Bootstrap simple size: 5,000. The total effect of perceived socioeconomic decline on informal PH appears within brackets. *p < .05, **p < .01, ***p < .001.

Additionally, as indirect effects can exist in the absence of a significant total effect [69], we also examined the indirect effect of perceived economic threat on informal planned helping via empathic concern. Results showed that perceived economic threat was associated to increased empathic concern, which in turn was related to greater scores on informal planned helping; this confirms that perceived economic threat was also indirectly linked to informal planned helping (*b* = .20, *SE* = .12, 95%CI [0.03, 0.52]) via its effect on empathic concern (see Fig 4). Moreover, because neither the total (*b* = .91, *SE* = .49, *p* = .064, 95%CI [-0.05, 1.87]) nor the direct (after controlling for empathic concern; *b* = .71, *SE* = .49, *p* = .147, 95%CI [-0.25, 1.68]) effects or perceived economic threat were significant, the mediation analysis yielded an indirect effect in absence of a significant total effect.

In short, results revealed that one reason that individuals who have descended the social ladder may be likelier to use the three types of helping behavior is related, in part, to their higher levels of empathic concern.

### Discussion

Study 1 offers initial empirical evidence supporting our main expectations. Our data consistently indicated an enhanced tendency to act prosocially among those participants who were perceived as having descended the social ladder, due to the Spanish national economic situation. In particular, the present study confirmed that perceived economic threat (i.e., perceived socioeconomic descent) was related to higher levels of two different types of helping behavior (i.e., formal planned helping behavior and spontaneous helping behavior). It is important to note that these effects emerged even after accounting for ideological (e.g., political orientation) and objective socioeconomic factors (e.g., employment status). In support of our mediational hypothesis, our data suggested that individuals who descended the social ladder due to the economic downturn reported increased helping behavior (i.e., formal and informal planned helping behavior, and spontaneous helping behavior) because of their elevated other-oriented feelings of sympathy and compassion (i.e., empathic concern). Altogether, this first study provides preliminary support for the idea that perceived economic threat is not always related to deleterious psychological responses [20], but it also leaves open the following question: Do people under considerable perceived economic threat show a generalized prosocial inclination or, conversely, display these prosocial expressions only toward others similar to them?

## Study 2

Study 2 aimed to shed light on the question formulated above in a large and diverse community sample. Thus, in this study we tested whether the effects on prosocial tendencies of personal economic threat related to the unfavorable Spanish economic context could vary as a function of the specific characteristics of the person potentially in need of help. A recent investigation conducted with Spanish undergraduate students [37] revealed that the salient lack of personal control related to unemployment led participants to endorse more solidarity with people facing similar economic situations (e.g., Greeks) because of the students’ greater identification and perceived similarity with them. Accordingly, we expected to find that perceptions of economic threat predicted higher levels of prosocial behavior toward similar others. Moreover, we predicted that this potential effect would be explained—at least in part—by enhanced empathic concern and identification. To test these specific predictions, we asked participants to indicate their willingness to help in response to two different scenarios describing one’s person suffering. In one case, the person’s suffering was linked to the national negative economic context, whereas in the other case it was not related to any type of economic condition. In addition to their desire to help, participants were also asked to indicate their empathic concern-related feelings toward and identification with the person after being exposed to the corresponding scenario.

### Method

#### Participants

Five hundred eighty-eight individuals (*M*_age_ = 33.42, *SD* = 10.02, range from 18 to 63) participated in this study. Sixty-eight percent of participants were women and 32% were men. More sociodemographic characteristics are detailed in Table 1. A sensitive power analysis was conducted using the fixed model R increase in G*Power [60] to determine our ability to detect the contribution of 2-way interactions in a multiple regression. Given our sample (*N* = 588, α = 0.05), sensitivity analysis suggests the need for a minimum effect size of *f*^2^ ≥ 0.01 to produce power at the 0.80 level.

#### Procedure

An online administration procedure was used, so respondents completed an online booklet that included the measures of interest. As in Study 1, the sample was obtained by means of an incidental sampling procedure. More specifically, participants were recruited via online advertisements (about the possibility of collaborating in a psychological research study on social issues) on Internet forums and social networks (e.g., Facebook). Advertisements were linked to the online survey. First, and as in the preceding study, individuals were given information guaranteeing their anonymity and confidentiality, as well as indicating the estimated duration for completion of the online survey (approximately 10 min). Then, those individuals who fulfilled the selection criterion—to have Spanish as native language—and agreed to participate in the study signed an informed consent form. Participants were randomly assigned to one of two conditions, in which they were asked to read a hypothetical helping behavior scenario—following a between-subjects design with two conditions (crisis-related helping scenario vs. control helping scenario, described below). After reading the corresponding hypothetical scenario, they were asked to complete our measures of interest. Next, participants were asked to fill out a standard demographic questionnaire. Finally, respondents entered a 50€ prize drawing for their participation. Upon completion of the study booklet, participants were thanked and debriefed. The study received approval from the institutional research ethics committee of the University of Granada (Number: 1002/CEIH/2019). All participants gave informed written consent in accordance with the Declaration of Helsinki.

#### Measures

*Helping behavior scenarios*. A portion of the participants read a scenario about a person who had a sign that indicated she/he lost her/his job, coinciding with the context of economic crisis, and had serious difficulties making ends meet (crisis-related helping scenario). The other participants were asked to read a text describing a person who had lost her/his wallet (control helping scenario). After reading the applicable text, all participants answered two manipulation checks, “To what extent is the situation of the described person due to the economic crisis?” and “To what extent is the situation of the described person due to a specific event?”, with a 7-point Likert-type response format ranging from 1 (*not at all*) to 7 (*too much*). The scenarios presented are provided in the S1 Appendix.

*Helping behavior*. Consistent with Ministero, Poulin, Buffone, and DeLury [70] (2018), we administered the Cameron and Payne [71] 4-item measure to evaluate participants’ helping behavior inclination. Participants answered this scale (e.g., “To what extent do you feel it is appropriate to give money” [to the person described in the scenario]; α = .84) on a 7-point Likert scale, ranging from 1 (*not at all*) to 7 (*extremely*).

*Empathic concern*. To evaluate empathic-related feelings toward the person described in each respective scenario, we used the 5-item measure originally developed by Cameron and Payne [71] and previously used by other researchers [70]. The responses to these items (e.g., “How touched do you feel” [toward the person described in the scenario]; α = .82) were measured on a 7-point Likert scale ranging from 1 (*not at all*) to 7 (*extremely*).

*Identification*. A single-item measure was used to assess the extent to which participants tended to identify with the person described in each respective helping behavior scenario (e.g., “To what extent do you identify with the person described”) and was measured on a 7-point Likert scale, ranging from 1 (*not at all*) to 7 (*extremely*).

*Perceived economic threat measures*. As in Study 1, we evaluated whether participants descended (or not) on the social scale due to the crisis [17]. Moreover, we also administered the Financial Threat Scale (FTS) [72]—adapted to the Spanish context of economic turmoil [18]—because it (1) overcomes the potential methodological limitations of the prior dichotomous measure and (2) directly assesses individuals’ psychological threats regarding their financial situations [18]. This 5-item instrument (e.g., “How much do you feel at risk”) has a 5-point Likert scale response format ranging from 1 (*not at all*) to 5 (*a great deal*; α = .89).

**Subjective social class, objective social class, and political orientation** were evaluated in the same manner as in Study 1.

#### Statistical analyses

First, two Student’s t-test were conducted to examine whether participants adequately differentiated the two helping behavior scenarios (1 = crisis-related scenario; 2 = control scenario). Second, and as a preliminary check for regression analyses, our predictor variables were standardized to determine that collinearity statistics were within acceptable values [73]. Afterward, we performed a hierarchical regression analysis to test the unique predictive contribution of perceived economic threat measures (i.e., perceived socioeconomic decline and perceived financial threat) to prosocial behavior (i.e., willingness to help) while controlling for the influence of sociodemographic and ideological characteristics (i.e., gender, objective and subjective socioeconomic status, employment status, and political orientation) and the type of helping scenario. Sociodemographic and ideological factors were entered in Step 1 (method: enter), perceived economic threat measures and the type of helping scenario were included in Step 2 (method: enter), and second order interactions were incorporated in Step 3 (method: enter) of the regression model. Third, we examined the indirect effect of perceived economic threat on helping behavior parallel to the empathic concern and identification with the person described in each helping behavior scenario. The analysis of the mediation model was performed using Hayes’ [68] PROCESS macro (Model 4) for SPSS statistical package. Sociodemographic and ideological characteristics (i.e., gender, objective and subjective socioeconomic status, unemployment status, and political orientation) were included as covariates. Lastly, we further verified whether these potential mediation effects were moderated by the type of helping behavior scenario. Moderated mediation is often used to scrutinize whether the magnitude of a mediation effect is conditional on the value of a moderator [68]. The analysis of the proposed moderated mediation model was performed using Hayes’ [68] PROCESS macro (Model 59) for SPSS statistical package. We estimated 95% bias-corrected confidence intervals for indirect associations based on 5,000 bootstrap samples. Confidence intervals that did not contain zero indicated that effects were significant (*p* < .05).

### Results

#### Manipulation check

Two Student’s t-test (between-subjects) analyses were conducted involving an independent variable with two levels (helping behavior scenario: crisis-related and control helping scenarios), and the manipulation check items as dependent variables. As expected, the situation of the person described in the crisis-related helping scenario was perceived to be more linked to the economic crisis (*M* = 5.22, *SD* = 1.44) in comparison with the control helping scenario (*M* = 3.29, *SD* = 1.95), *t* = 13.60, *p* < .001, *d* = 1.13. Furthermore, the control helping scenario was perceived to be more related to a one-off occurrence (*M* = 5.31, *SD* = 1.73) than the crisis-related helping scenario (*M* = 3.67, *SD* = 1.71), *t* = -11.53, *p* < .001, *d* = -0.95. These outcomes suggest that participants adequately differentiated the two proposed helping behavior scenarios.

#### Testing the unique contribution of perceived economic threat measures to prosocial behavior

Table 3 displays the results from the hierarchical multiple regression analysis predicting the willingness to help from sociodemographic and ideological characteristics (i.e., gender, objective and subjective socioeconomic status, unemployment status, and political orientation), helping scenarios, and perceived threat measures (i.e., perceived socioeconomic decline and perceived financial threat).

**Table 3:** Hierarchical regression analysis predicting helping behavior (Study 2).

Predictors	*ΔR*^*2*^	β	*t*	CI (95%)
Step 1	.07***			
Gender		-.16***	-3.93	[-.070, -0.23]
Objective SES		-.05	-1.12	[-0.25, 0.07]
Subjective SES		-.09	-1.84	[-0.24, 0.01]
Employment status		.05	1.20	[-0.10, 0.39]
Political orientation		-.12**	-2.86	[-0.27, -0.05]
Step 2	.03***			
Helping scenarios		-.12**	-3.05	[-0.54, -0.12]
Socioeconomic decline		-.01	-0.19	[-0.27, 0.22]
Perceived financial threat		.15**	3.08	[0.07, 0.32]
Step 3	.01			
Socioeconomic decline x helping scenarios		-.27	-1.35	[-0.80, 0.15]
Perceived financial threat x helping scenarios		-.04	-0.29	[-0.27, 0.20]
Socioeconomic decline x perceived financial threat		.09	0.66	[-0.15, 0.31]

*N* = 588; SES: Socioeconomic status; Gender (1 = males, 2 = females); Employment status (1 = no unemployed, 2 = unemployed); Socioeconomic decline (1 = no, 2 = yes); Helping scenarios (1 = crisis-related scenario, 2 = control scenario).

** *p* < .01,

*** *p* < .001

Results indicated that sociodemographic and ideological factors entered in Step 1 together accounted for 0.068% of the variance in helping behavior, *F*(5,556) = 8.11, *p* < .001. In particular, female gender (β = -.16, *t* = -3.93, *p* < .001, 95% CI [-0.70, -0.23]) and liberal political ideology (β = -.12, *t* = -2.86, *p* = .004, 95% CI [-0.27, -0.05]) predicted a greater willingness to help.

When the type of helping scenario and perceived economic threat indicators were added at the second step, the regression model remained significant, *F*(8,553) = 7.66, *p* < .001. The inclusion of these variables in the regression equation explained an additional 3.2% of the variance in helping behavior, *F*(3,553) = 6.52, *p* < .001. As illustrated in Table 3, the type of helping scenario significantly predicted participants’ willingness to help, (β = -.12, *t* = -3.05, *p* = .002, 95%CI [-0.54, -0.12]), indicating a higher likelihood to help in the crisis-related scenario (vs. control scenario). Interestingly, although we did not find differences in the levels of the willingness to help based on whether participants descended (or not) on the social scale, (β = -.01, *t* = -0.19, *p* = .849, 95%CI [-0.27, 0.22]), our results showed that perceived financial threat related to the economic crisis contributed to the prediction of desire to help, (β = .15, *t* = 3.08, *p* = .002, 95% CI [0.07, 0.32]). Elevated perceived financial threat was indicative of a greater tendency to help, even after accounting for individuals’ sociodemographic and ideological characteristics and the type of helping scenario.

Importantly, the incorporation of the interaction terms between perceived threat measures and helping behavior scenarios in the next step did not significantly explain an additional proportion of the variance in helping behavior, *F*(3,550) = 1.01, *p* = .34. None of the second order interaction effects emerged as significant (all *ps* >.05; Table 3).

#### Testing the indirect contribution of perceived financial threat to prosocial behavior via empathic concern and identification

Considering the findings of Study 1 and the significant predictive contribution of the perceived financial threat indicator to helping behavior, we analyzed the potential mediation effects of empathic concern and identification within the perceived financial threat-helping behavior association.

**Fig 5:**
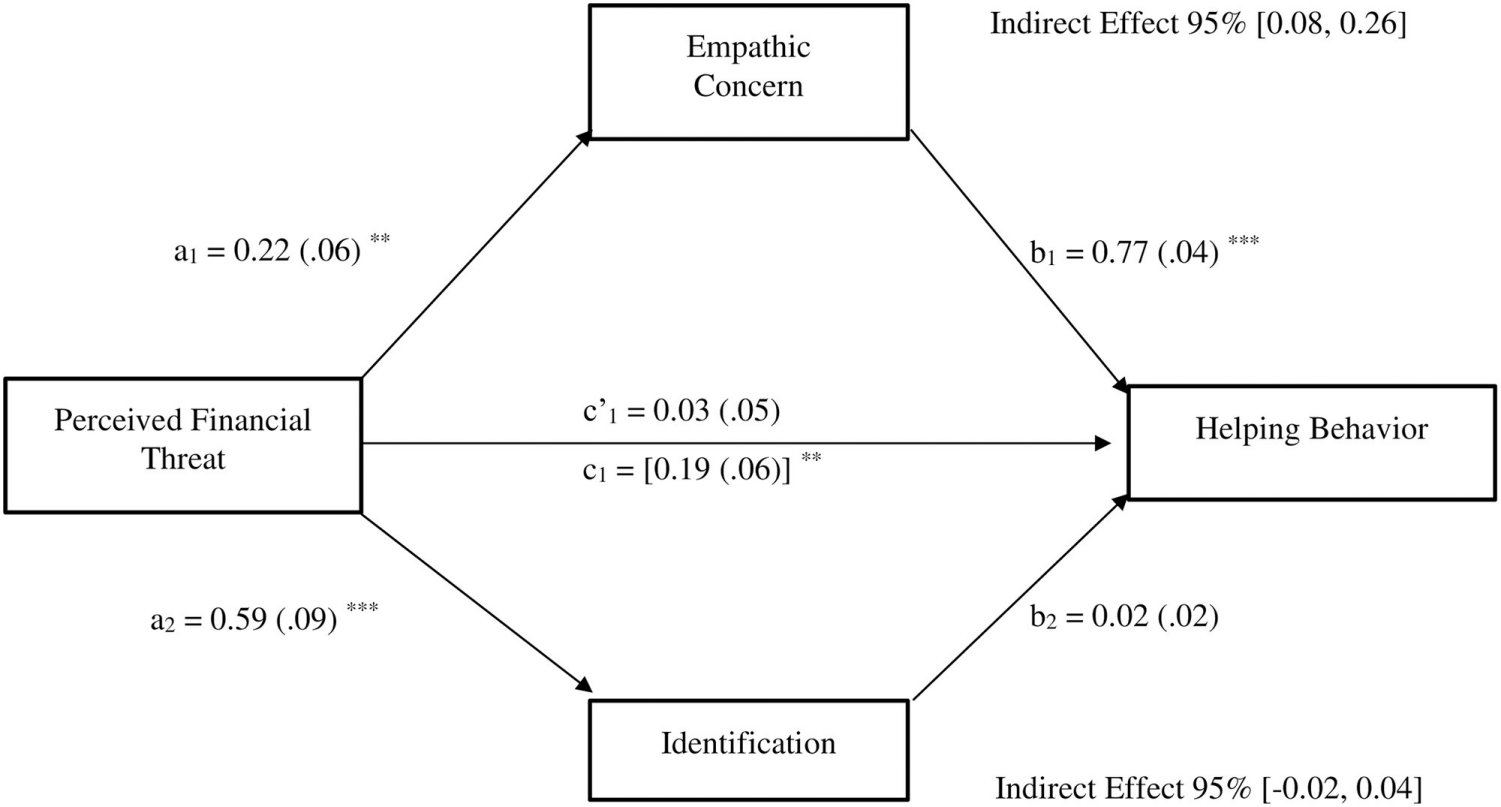
Parallel mediation model depicting the indirect effect of perceived financial threat on helping behavior through empathic concern and identification. Study 2; *N* = 588. All reported values are unstandardized estimates (*b* values), with their SE reported between parentheses. Bootstrap simple size: 5,000. The total effect of perceived financial on helping behavior appears within brackets. *p < .05, **p < .01, ***p < .001.

Perceived financial threat was significantly associated with desire to help (*b* = .19, *SE* = .06, 95% CI [0.08, 0.31]; see Fig 5c_1_). Moreover, as Fig 5 shows, the associations between perceived financial threat and empathic concern (a_1_), and between empathic concern and willingness to help (b_1_) were significant. Likewise, aligning with our expectations, results showed that perceived financial threat was also indirectly linked to the desire to help (*b* = .17, *SE* = .05, 95% CI [0.08, 0.27]) via its effect on empathic concern. The direct effect of perceived financial threat on the desire to help was not significant after controlling for the mediator variable (i.e., empathic concern, *b* = .03, *SE* = .05, *p* = .534, 95% CI [-0.07, 0.13]; see Fig 5c’_1_), confirming in this case the existence of a complete mediation. Conversely, although the association between perceived financial threat and identification toward the person described in the scenarios was significant (a_2_), the association between identification and the desire to help was not significant (b_2_), indicating in this case the absence of mediation (see Fig 5). Therefore, perceived financial threat was not indirectly linked to desire to help (*b* = .01, *SE* = .02, 95% CI [-0.02, 0.04] via its effect on identification.

In short, individuals who reported increased financial threat showed higher empathic concern and identification toward the person described in the scenarios. However, only the higher levels of empathic concern were, in turn, related to greater scores on the desire to help.

#### Testing the moderated mediation effects of helping behavior scenarios

**Fig 6:**
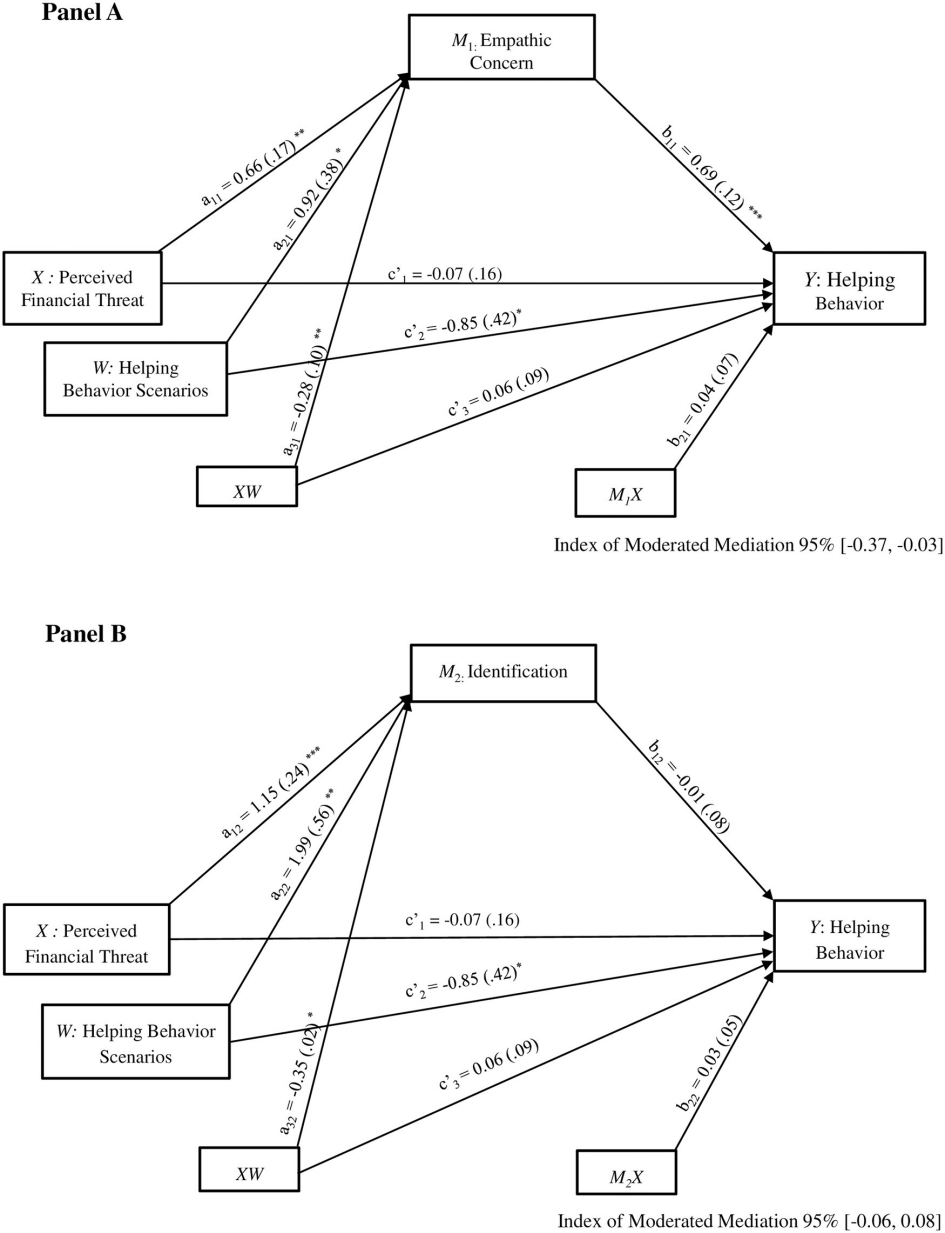
Parallel moderated mediation model depicting the indirect effect of perceived financial threat on helping behavior through empathic concern (panel A) and identification (panel B). Study 2; *N* = 588. All reported values are unstandardized estimates (*b* values), with their SE reported between parentheses. Bootstrap simple size: 5,000. *p < .05, **p < .01, ***p < .001.

We turned next to extend those prior results by analyzing whether the potential mediation effects of empathic concern and identification were significant for each helping behavior scenario (*a mediated moderation*). We estimated parameters (parallel) for three regression models [74,75], that is, the moderating effect of helping scenarios on (1) the association of perceived financial threat with (a) empathic concern and (b) identification (first stage moderation); (2) the relationship of (a) empathic concern and (b) identification with helping behavior (second stage moderation); and (3) the relationship between perceived financial threat and desire to help accounting for the mediator variables’ effects (direct effect moderation). We controlled for sociodemographic and ideological characteristics (i.e., gender, objective and subjective socioeconomic status, employment status, and political orientation). Results are presented in Fig 6; panel A displayed the moderated mediation with empathic concern as mediator and panel B displayed the moderated mediation with identification as mediator.

**Fig 7:**
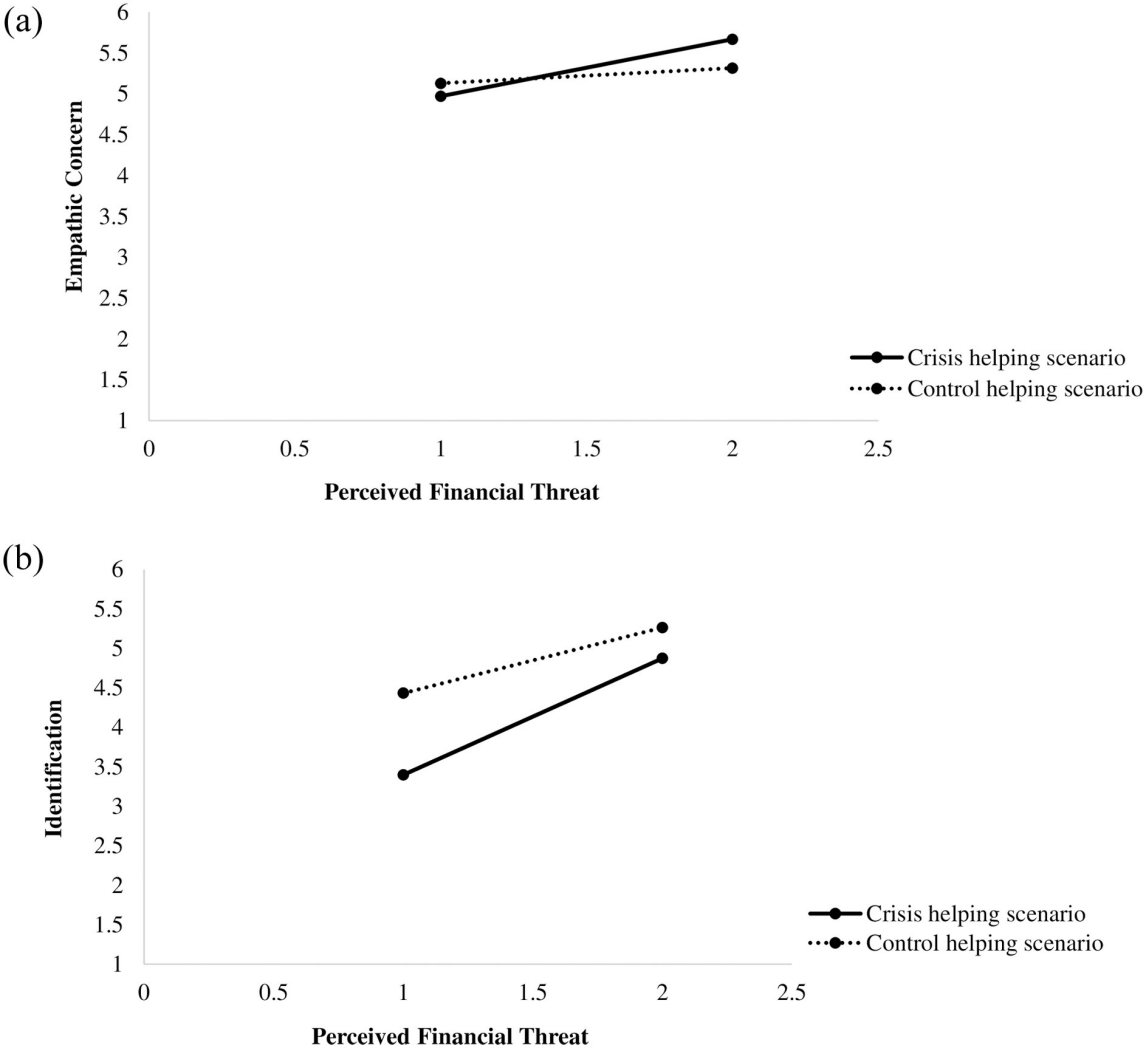
**A.** Individuals’ empathic concern levels as a function of perceived financial threat and helping behavior scenarios. **B.** Individuals’ identification levels as a function of perceived financial threat and helping behavior scenarios.

As Fig 6 illustrates, results showed a significant effect of perceived financial threat on both (a) empathic concern (see Fig 6; panel A; a_11_) and (b) identification (see Fig 6; panel B; a_12_); moreover, the type of helping scenario moderated each of these effects (a_31_, a_32_, respectively). Simple slope tests indicated that a higher personal financial threat was associated with an increased empathic concern toward the person described in the crisis-related helping scenario (*b*_simple_ = .38, *SE* = .08, *p* < .001, 95% CI [0.22, 0.54]). However, a significant association between perceived financial threat and empathic concern toward the person described in the control helping scenario did not emerge (*b*_simple_ = .10, *SE* = .07, *p* = .161, 95% CI [−0.04, 0.24]; see Fig 7A).

Regarding the identification variable, simple slope tests indicated that higher levels of perceived financial threat were associated with an increased identification, both with the person described in the crisis-related helping scenario and with the one described in the control helping scenario; however, it is important to mention that the strength of the *b* coefficient was higher for the perceived financial threat-identification relationship in the crisis-related helping scenario (*b*_simple_ = .80, *SE* = .12, *p* < .001, 95% CI [0.57, 1.03]), as compared to the control helping scenario (*b*_simple_ = .45, *SE* = .11, *p* < .001, 95% CI [0.25, 0.66]; see Fig 7B).

**Fig 8:**
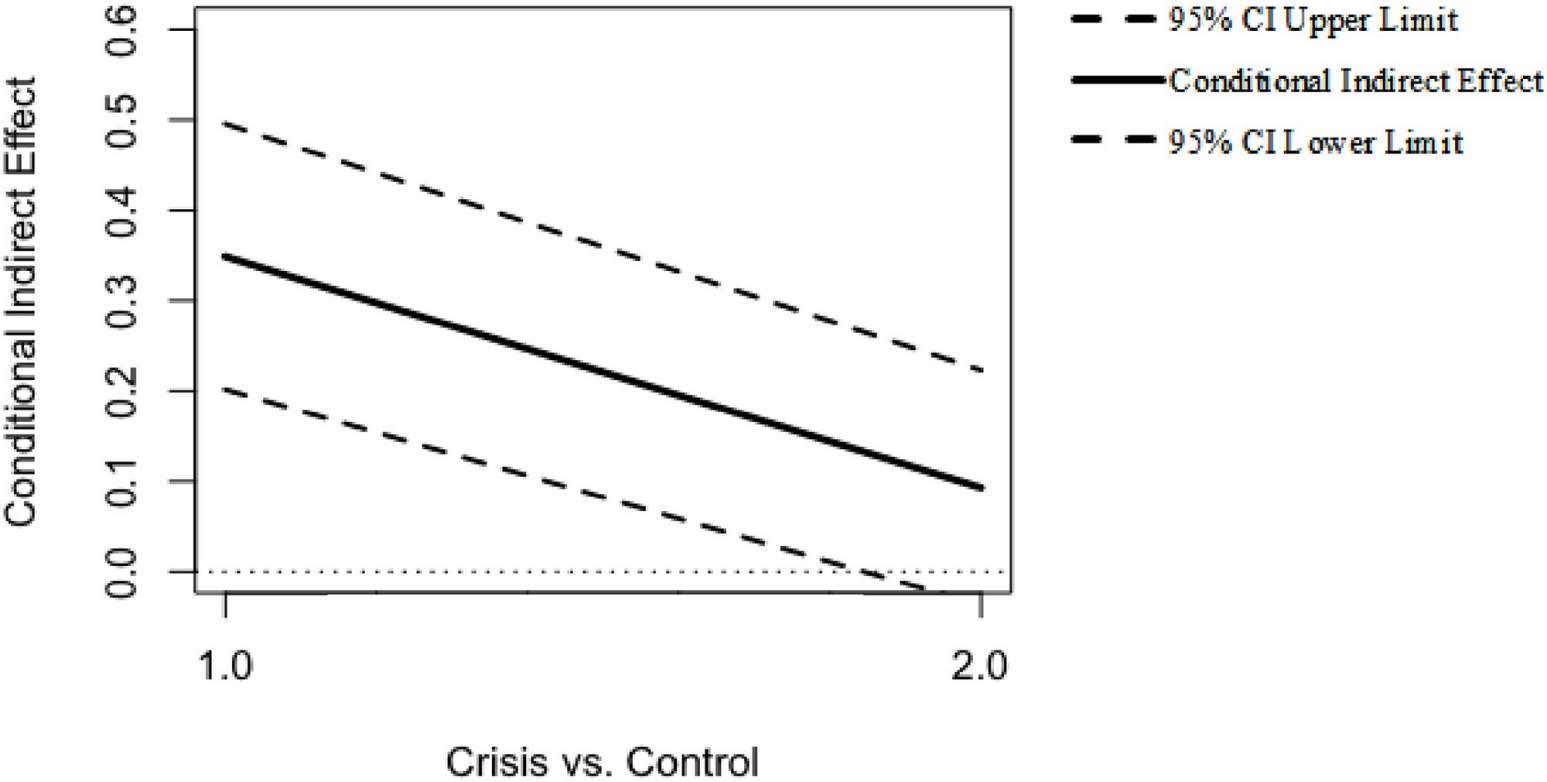
Conditional indirect effect of perceived financial threat on helping behavior at values of the moderator helping behavior scenarios through empathic concern.

Second, results yielded a significant effect of empathic concern on the desire to help (b_11_); this effect was not moderated by the type of helping behavior scenario (b_21_). Conversely, identification was not associated with the desire to help (b_12_), nor was this effect moderated by the type of helping scenario (b_22_). Finally, the direct effect of perceived financial threat on desire to help was not significant after controlling for the mediators’ variables (c’_1_); this effect was not moderated by the type of helping scenario (c’_3_). Therefore, individuals who reported increased financial threat showed higher empathic concern (in the crisis-related scenario) and identification with the person described in both scenarios (crisis-related and control). However, only heightened empathic concern was, in turn, related to greater scores on desire to help. These results were confirmed by the index of moderated mediation that represents the slope of the line for the association between the moderator (i.e., type of helping scenario) and the indirect effect of personal economic threat on the desire to help [68]. The bias-corrected percentile bootstrap for this index indicated that there was not an indirect effect of perceived financial threat on helping behavior through identification moderated by the type of helping scenario (*b* = .01, *SE* = .04, 95% CI [−0.06, 0.08]). Importantly, the index of moderated mediation corroborated that the indirect effect of perceived financial threat on desire to help because of empathic concern was moderated by the type of helping scenario (*b* = −.20, *SE* = .09, 95% CI [−0.37, −0.03]). Specifically, in the crisis-related helping scenario, a higher perception of financial threat was linked to a greater desire to help through an individual’s increased empathic concern (*b* = .28, *SE* = .07, 95% CI [0.16, 0.43]). In contrast, the indirect effect was not significant for the control helping scenario (*b* = .08, *SE* = .06, 95% CI [−0.04, 0.20]; see Fig 8). Given that the type of helping scenario only moderated the first stage of the mediation process, the results showed evidence of a “first stage moderation model,” which is one form of moderated mediation model.

### Discussion

Using two different helping behavior scenarios, Study 2 indicated that participants with higher scores on perceived financial threat reported greater levels of willingness to help. This finding is consistent with the one observed in the preceding study, which showed significant positive relationships of perceived economic threat with various measures of helping behavior. In addition, our results confirmed that such effects were not only independent of the same sociodemographic and ideological variables that were controlled for in Study 1 but also of the type of helping behavior scenario (crisis-related vs. control) considered. Interestingly, but contrary to our main hypothesis, this observation seems to suggest that being affected by a strained economic situation does not necessarily have to be linked to greater prosocial behavior toward others who are similar (i.e., those facing the negative consequences of the national economic situation). Rather, our data revealed that participants who scored high on perceived financial threat exhibited an undifferentiated pattern of prosociality. Stated another way, they showed generalized others-oriented behavioral inclinations. Additionally, Study 2 findings allowed us to determine that the effect of perceived financial threat on helping behavior was driven by enhanced empathic concern in the crisis-related helping behavior scenario but not in the control scenario, thus extending the results of Study 1. Also, it is worth noting that identification did not emerge as a significant mediator of the perceived financial threat-helping behavior association in any of the two proposed scenarios. Therefore, although perceived financial threat predicted an increased and generalized prosocial tendency, this study suggests that the mechanisms (e.g., empathic concern) that might account for this effect could indeed differ in terms of the particular features of the target person.

## General discussion

Even though the socioeconomic and political impact of the global economic downturn has been extensively examined, the psychosocial effects of the perceived economic threat linked to this period of widespread economic enfeeblement remains undoubtedly elusive. To address this gap, and to extend prior empirical evidence [16, 18] by focusing on prosociality, we tested, across two independently collected community samples, whether (a) a higher perceived economic threat linked to the negative Spanish economic situation is connected to a greater inclination to engage in other-beneficial prosocial behavior, and (b) this relationship could be explained by empathic concern and identification.

In one set of analyses (Study 1), we found that participants who descended on the social scale due to the unfavorable Spanish economic situation showed greater scores for various forms of helping behavior (i.e., formal planned helping and spontaneous helping) in comparison with those who did not descend in the social class hierarchy. Importantly, we noted that these effects remained significant even after controlling for several sociodemographic and ideological potential confounds known to be associated with other-oriented emotions and behaviors. These findings advance our understanding of how the perceived personal impact of a tough economic climate may influence prosocial behavior independently of an individual’s material circumstances (e.g., socioeconomic status). Thus, our data support, at the individual level, prior research suggesting that socioeconomic crises can be frequently accompanied by a rise in volunteering [36], and, moreover, the shared common idea that those citizens who suffer most from the negative impact of these types of economic contexts are more willing to participate in mutual social assistance movements, probably as a means of facing the negative consequences [19, 76,77]. This tendency to affiliate with others or join social groups, also called “befriending,” might finally help individuals build a sustained cooperative network to defend their own communities from social threats [27,28].

Study 1 also consistently showed that the effects of perceived socioeconomic descent on prosocial behavior were explained by enhanced empathic concern. Therefore, another contribution of our findings is the identification of an important emotional mechanism that drives the indirect perceived economic threat-other-beneficial behavior link. Specifically, our results suggest that feeling adversely affected by the economic downturn may promote being more emphatically concerned about others’ suffering, which in turn may positively affect different types of prosocial behavior. This pattern of results appears to indicate that individuals who are undergoing hard life situations—such as the effects of the national economic decline—might feel greater vulnerability to others in the social environment [34, 78]. This exposure to socioeconomic threats involves a heightened vigilance that could predispose people affected by the economic crisis to focus their attention on the context and, in particular, on other individuals as a possible way to understand events in their lives [29]. As a result, individuals may orient to other people and, presumably, should be more aware of others’ emotions to navigate their surrounding adverse socioeconomic conditions. In other words, those individuals most affected by the economic downturn may exhibit higher empathic concern for the welfare of others, in order to alleviate negative personal feelings by promoting greater attention to the suffering of others [28, 79,80]. Moreover, empathic concern was related to a higher likelihood of adopting prosocial behaviors. Therefore, this finding extends previous evidence on the relation between empathy and prosocial behavior [38, 49–53] by showing its association in people who undergo the effects of the economic crisis.

Study 2 also provides noteworthy findings. In this study, through the administration of two different helping behavior scenarios (crisis-related scenario vs. neutral scenario), our results indicated that individuals high in perceived financial threat were more prone to help, even after accounting for sociodemographic and ideological factors and the type of helping scenario. This last finding is particularly important because it reflects that a participant’s willingness to help did not differ based on the similarity of the person described in the helping behavior scenarios, thus supporting the unexpected notion that citizens most affected by the Spanish economic situation tend to show a nonspecific other-beneficial behavioral pattern. In spite of this, it should also be stated that, when we account for the indirect mechanisms (empathic concern and identification) that could explain the effect of perceived economic threat on prosocial behavior, the results of Study 2 showed more intense effects of perceived financial threat on identification and empathic concern toward the person described in the crisis-related helping behavior scenario. These results are conceptually similar to those deriving from prior studies, revealing greater identification and similarity with other individuals who are affected by unemployment and higher in-group bias under salient threat of personal control [37, 81]. Interestingly, further moderated-mediation analyses confirmed that empathic concern, but not identification, mediated the effects of perceived financial threat on the willingness to help to the person described in the crisis-related scenario (the one who faced serious economic difficulties linked to the economic downturn). This result reveals that, even though participants high in perceived financial threat tended to identify more strongly with the person described in the crisis-related scenario, this process of identification would not trigger greater helping behavior per se. Rather, our data indicated that feelings of empathic concern toward such a person would tip the balance toward helping. Thus, empathic concern seems to represent a crucial condition for eliciting helping behavior toward individuals who are in similar negative situations [34].

Consistent with our findings, psychologists have strongly reinforced the idea that empathy is a major determinant of prosociality [38, 51, 82]. However, because identification alone would not engender prosocial behaviors, this research highlights the need to consider how identification processes could contribute to the willingness to help. While identification between the self and others has also been shown to facilitate cooperative behaviors [83], recent findings stress the importance of intergroup identification for promoting empathy-based reactions [84,85]. Thus, the positive effects of empathy could occur as a result of the mere activation of a communal orientation by enhancing identification with one’s in-group and distinction from one’s out-group [86,87]. Therefore, as Oveis, Horberg, and Keltner [88] suggested, it is plausible that emotions that guide prosocial actions evolved alongside powerful cognitive intuitions, such as the identification or similarity to others, attention to harm, and the fairness perception with respect to the allocation of resources. Overall, our findings contribute to illuminate the underlying mechanism (e.g., empathic concern) involved in the perceived financial threat-helping behavior link in the crisis-related scenario. In this regard, it should be remarked that our results support a generalized prosocial inclination among those showing greater levels of perceived financial threat. However, our data do not provide support for the mediating role of either identification or empathic concern in the perceived financial threat-desire to help relation within the neutral helping behavior scenario (the one that describes a relatively common suffering-related event). Therefore, further research is needed to clarify which emotional and cognitive intervening variables—and the interdependences between them—operate in such particular conditions.

### Limitations and avenues for future research

The two independent studies presented have certain limitations that should be acknowledged, while proposing potential avenues to explore in subsequent studies. Although our research includes large and heterogeneous community-based samples, data was derived from non-probabilistic sampling, potentially constraining the generalization of the results obtained. In the same vein, the employed nonexperimental methodology hampers the possibility of establishing the causal connection of the associations studied. Thus, it would be advisable to conduct longitudinal designs to ascertain how changes over time in people’s perceived economic threat levels (as well as in objective socioeconomic factors) affect their prosocial tendencies. Moreover, given that the two helping behavior scenarios tested in Study 2 are essentially money-related, thus sharing an underlying nature, subsequent studies should incorporate at least one additional and distinctive scenario (e.g., depicting a person suffering from major health problems) to provide a more complete picture of differences in helping behavior tendencies. Further research could also provide substantive evidence that complement and strengthen our central findings by analyzing whether the effects of personal economic threat are modulated by, for example, macro-social variables (e.g., social inequality, economic growth, etc.). Lastly, it is worth mentioning that our main assumptions were tested in one of the European countries most negatively affected by the economic crisis; however, it may be desirable to obtain additional empirical evidence in other countries still experiencing serious economic difficulties.

### Concluding remarks

This research offers preliminary, consistent, and valuable empirical evidence that those individuals with higher levels of perceived economic threat, linked to the tough Spanish economic context, are more inclined to exhibit prosocial behavior. In addition, our results indicated that empathic concern represents a plausible psychological mechanism through which perceived personal impact of the economic crisis relates to prosocial behavior toward (similar) others in times of economic hardship. In short, the present investigation modestly provides insight into the growing research on the effects of subjective economic experiences on psychological outcomes.

## Supporting information

S1 Appendix Helping behavior scenarios. (DOCX)
