# Supplementary material for: Connecting perceived economic threat and prosocial tendencies: The explanatory role of empathic concern
Source: PLoS One. 2020 May 4;15(5):e0232608. doi: 10.1371/journal.pone.0232608 (PMC7197816; doi:10.1371/journal.pone.0232608)
Supplement: S1 Appendix — (DOCX) [file pone.0232608.s001.docx]

**Supporting information**

**S1 Appendix. Helping behavior scenarios.**

**Helping behavior scenarios**

Crisis-related scenario:

At about 8:30 p.m. after a long day, you arrive at the bus stop that will take you home. A few meters from the stop, you see a person sitting on a bench. This person seems to be having a hard time because he/she is crying. As you approach, you notice that he/she has a sign that says that, coinciding with the economic crisis, he/she lost his/her job and has serious problems making ends meet.

Control scenario:

At about 8:30 p.m. after a long day, you arrive at the bus stop that will take you home. A few meters from the stop, you see a person sitting on a bench. This person seems to be having a hard time because he/she is crying. As you approach, you realize that he/she is saying that he/she cannot find his/her wallet.
